# Comprehensive Analysis of DMRT3 as a Potential Biomarker Associated with the Immune Infiltration in a Pan-Cancer Analysis and Validation in Lung Adenocarcinoma

**DOI:** 10.3390/cancers14246220

**Published:** 2022-12-16

**Authors:** Donghong Yang, Meilian Liu, Junhong Jiang, Yiping Luo, Yongcun Wang, Huoguang Chen, Dongbing Li, Dongliang Wang, Zhixiong Yang, Hualin Chen

**Affiliations:** 1 Department of Oncology, Affiliated Hospital of Guangdong Medical University, Zhanjiang 524000, China; 2 Department of Pulmonary Oncology, Affiliated Hospital of Guangdong Medical University, Zhanjiang 524000, China; 3 Department of Medicine, ChosenMed Technology (Beijing) Co., Ltd., Beijing 100176, China

**Keywords:** pan-cancer, DMRT3, prognosis, genomic alterations, immune

## Abstract

**Simple Summary:**

Doublesex and Mab-3 related Transcription Factor 3 (DMRT3) is associated with the prognosis of some tumors. It is possible to explore the mechanisms and functions of the DMRT3 gene in the cancer process, using bioinformatic approaches and experimental validation. We comprehensively explored the clinical and immunological characteristics of DMRT3. We identified that DMRT3 was aberrantly expressed in pan-cancer and may promote tumorigenesis and progression via different mechanisms. DMRT3 can be used as a therapeutic target to treat cancer in humans.

**Abstract:**

Doublesex and Mab-3 related Transcription Factor 3 (DMRT3) is associated with the prognosis of some tumors. It is possible to explore the role of DMRT3 in the cancer process using bioinformatic approaches and experimental validation. We comprehensively explored the clinical and immunological characteristics of DMRT3. The DMRT3 expression is abnormal in human cancers and correlates with clinical staging. A high DMRT3 expression is significantly associated with poor overall survival (OS) in KIRC, KIRP, LUAD, and UCEC. Amplification was the greatest frequency of the DMRT3 alterations in pan-cancer. The OS was significantly lower in the DMRT3 altered group than in the DMRT3 unaltered group (P = 0.0276). The DMRT3 expression was significantly associated with MSI in three cancer types and TMB in six cancer types. The DMRT3 expression was significantly correlated with the level of the immune cell infiltration and the immune checkpoint genes. The DMRT3 was involved in some pathways in pan-cancer. DMRT3 may play a role in chemotherapy and may be associated with chemoresistance. A ceRNA network of KCNQ1OT1/miR-335-5p/DMRT3 was constructed in LUAD. DMRT3 was significantly upregulated in the LUAD cell lines. DMRT3 was aberrantly expressed in pan-cancer and may promote tumorigenesis and progression via different mechanisms. DMRT3 can be used as a therapeutic target to treat cancer in humans.

## 1. Introduction

Globally, cancer is the leading cause of death and seriously reduces people’s chances of surviving [1]. Lung adenocarcinoma (LUAD) is the leading cause of cancer-related deaths worldwide and is the most common histologic subtype, accounting for approximately 40% of lung cancer incidences [2,3]. Many types of cancer still have an unsatisfactory prognosis and survival rates, despite significant advances in treatment in recent years [4]. Cancer immunomodulation contributes to the tumor growth, progression, and tumor microenvironment, as well as to the patient prognosis [5]. In light of the enormous potential of the tumor immune microenvironment (TIME) for cancer therapy, further investigation of the underlying mechanisms and the identification of new key biomarkers is urgently required.

The Doublesex and Mab-3 related Transcription Factor 3 (DMRT3) is a member of the DMRT family, involved in determining the sexual dimorphism and reproducing sexually [6,7]. DMRT3 is significantly overexpressed in the nasal polyps of patients with respiratory disease (AERD) [8]. There is, however, no understanding of how DMRT3 plays a role in pan-cancer.

In the present study, based on the expression, predictive value, clinicopathology, genetic alterations, GO (Gene Ontology) and KEGG (Kyoto Encyclopedia of Genes and Genomes), TME, immune-related genes, immune infiltration, MSI, TMB, and drug sensitivity in a variety of cancer types, we conducted a systematic analysis. Our findings suggest that DMRT3 may be of potential value in the tumor diagnosis and prognosis and may be used as a marker for immunotherapy.

## 2. Materials and Methods

### 2.1. Analysis of the DMRT3 Expression in Pan-Cancer

The DMRT3 mRNA expression map was constructed by the Human Protein Atlas (HPA) database (version: 20.1).

It was investigated whether tumor and non-tumor tissues in various tumor types express DMRT3 differently, using the “Gene DE” module of TIMER (http://timer.cistrome.org/ (accessed on 15 December 2022).

R (Version 3.6.3) and ggplot2 [version 3.3.3] (R Core Team, Vienna, Austria) were used for the statistical analysis and visualization [9]. The molecule was DMRT3 [ENSG00000064218]. Transcripts per million reads (TPMs) were derived from UCSC XENA (http://xenabrowser.net/datapages/) (accessed on 15 December 2022). RNAseq data from The Cancer Genome Atlas (TCGA) and GTEx, which were processed uniformly by the Toil process [10,11]. RNAseq data, in TPM format and log2, were transformed for the comparative analysis. The data were not filtered. The subgroups were not filtered. The subgroups include ACC, BLCA, BRCA, CESC, CHOL, COAD, DLBC, ESCA, GBM, HNSC, KICH, KIRC, KIRP, LAML, LGG, LIHC, LUAD, LUSC, MESO, OV, PAAD, PCPG, PRAD, READ, SARC, SKCM, STAD, TGCT, THCA, THYM, UCEC, UCS, and UVM [12].

### 2.2. Correlation Analysis of DMRT3 and Prognosis in Pan-Cancer

The RNA-sequencing expression (level 3) profiles and corresponding clinical information for 33 tumors were downloaded from the TCGA dataset (https://portal.gdc.com) (accessed on 15 December 2022). [13,14]. The univariate Cox regression analysis and the forest were used to show the *p* value, HR, and 95% CI of each variable through the ‘forestplot’ R package. All analysis methods and the R package were implemented by R version 4.0.3 [15]. If not stated otherwise, the two-group data were performed using the Wilcox test [16]. The survival outcomes included the overall survival (OS), progression-free survival (PFS), and disease-specific survival (DSS). 

### 2.3. Correlation of the DMRT3 Expression with the Stage in Pan-Cancer 

From the GEPIA 2 website (http://gepia2.cancer-pku.cn/#index) (accessed on 15 December 2022), the DMRT3 expression data were obtained for cancers at different clinical stages [17].

### 2.4. Correlation of the DMRT3 Expression with TMB and MSI in Pan-Cancer

The RNAseq data (level3) and corresponding clinical information for 33 tumors were obtained from the TCGA database (https://portal.gdc.com) (accessed on 15 December 2022). The TMB and MSI were derived from [18,19]. The statistical analyses were performed using R software version 4.0.3 (R Core Team, Vienna, Austria) [20,21]. 

### 2.5. Genomic Alterations of DMRT3 in Pan-Cancer

Using cBioPortal (http://www.cbioportal.org/)(accessed on 15 December 2022), the TCGA pan-cancer dataset was analyzed for the genetic alterations of DMRT3 [22]. The genetic alterations and mutation sites of DMRT3 were obtained through the “Oncoprint”, “Cancer Type Summary” and “Mutations” modules.

### 2.6. Analysis of the Association between the DMRT3 Expression and the Tumor Immune Microenvironment in Pan-Cancer

The “Immunity” module of TIMER2 (http://timer.cistrome.org/)(accessed on 15 December 2022) was used to explore the correlation between the DMRT3 expression and the cancer-associated fibroblast infiltration using algorithms, such as the extended multidimensional immunome characterization (EPIC), microenvironmental cell population-counter (MCP-counter), cell type enrichment analysis (XCELL), and tumor immune dysfunction and exclusion (TIDE) algorithms, to explore the correlation between the DMRT3 expression and the cancer-associated fibroblast infiltration [23].

The RNAseq data (level 3) and corresponding clinical information for 33 tumors were obtained from the TCGA database (https://portal.gdc.com) (accessed on 15 December 2022). SIGLEC15, IDO1, CD274, HAVCR2, PDCD1, CTLA4, LAG3, and PDCD1LG2 are the transcripts associated with the immune checkpoints [24]. The expression values of these eight genes were extracted to observe the expression of the immune checkpoint-associated genes [24]. A statistical analysis was performed using R software version 4.0.3 (R Core Team, Vienna, Austria) [16]. 

### 2.7. DMRT3-Related Functional Enrichment Analysis

Proteins bound to DMRT3 were analyzed, based on the STRING website (version 11.5) (https://string-db.org/)(accessed on 15 December 2022). The following thresholds were set to obtain the bound proteins: network type as full network, active interaction source for the experiment, and a minimum required interaction score of low confidence (0.150). The significance of the network edges was set to evidence, while the maximum number of interactors displayed was set to no more than 50 interactors. We identified 17 proteins that bind to DMRT3. In addition, we analyzed genes with similar expression patterns to DMRT3 in pan-cancer by GEPIA2 and selected the top 100 genes as candidates. A GO enrichment analysis and KEGG pathway analysis of DMRT3, 17 binding proteins, and 100 candidate genes were performed on the DAVID website (https://david.ncifcrf.gov/)(accessed on 15 December 2022) [25].

### 2.8. Drug Sensitivity of DMRT3 in Pan-Cancer

The RNAactDrug database (http://bio-bigdata.hrbmu.edu.cn/RNAactDrug/index.jsp) (accessed on 15 December 2022) was used to perform a drug sensitivity analysis of DMRT3 in pan-cancer. The omics screening condition was “expression”. The top five significantly positively correlated drugs and the top five significantly negatively correlated drugs were counted according to Spearman’s correlation coefficient.

### 2.9. Single Cell Sequencing Data Analysis

CancerSEA (http://biocc.hrbmu.edu.cn/CancerSEA/home.jsp) (accessed on 15 December 2022) is a specialized single-cell sequencing database that provides different functional states of cancer cells at the single-cell level [26]. We downloaded the single cell sequencing data, based on CancerSEA, the correlations between the DMRT3 expression, and the different tumor functional states and plotted heat maps. The t-SNE maps of all individual cells were obtained directly from the CancerSEA website.

### 2.10. Conservation Analysis of DMRT3

The UCSC Genome Browser (version: 2021 update) (http://www.genome.ucsc.edu/cgi-bin/hgTracks) (accessed on 15 December 2022) was used to visualize the gene conversation of DMRT3 in vertebrates [23].

### 2.11. CeRNA Network Construction 

To explore the potential functions of DMRT3 in LUAD, we constructed a competitive endogenous RNA (ceRNA) network. Using miRTarBase (http://mirtarbase.cuhk.edu.cn/) and TarBase V.8 (https://carolina.imis.athena-innovation.gr/diana_tools/web/index.php?r= tarbasev8%2Findex) (accessed on 15 December 2022) to predict the miRNAs that bind to target genes [14,27]. Based on the miRNAs obtained from the screening, StarBase (http://starbase.sysu.edu.cn/) and LncBase Predicted v.2 (https://carolina.imis.athena-innovation.gr/diana_tools/web/index.php?r=lncbasev2/index-predicted) (accessed on 15 December 2022) were used to predict the lncRNAs interacting with miRNAs.

R version 3.6.3 and ggplot2 version 3.3.3 (R Core Team, Vienna, Austria) were used for the statistical analysis and visualization [22,28]. The molecules were hsa-miR-335-5p [MIMAT0000765], and KCNQ1OT1 [ENSG00000269821]. The data were miRNAseq data from the level 3 BCGSC miRNA Profiling in the TCGA LUAD project and the RNAseq data in the level 3 HTSeq-FPKM format from the TCGA LUAD project [9,28]. 

R version 3.6.3, survminer package version 0.4.9, and survival package version 3.2-10 (R Core Team, Vienna, Austria) were used for the statistical analysis and visualization [11]. The molecules were hsa-miR-335-5p and KCNQ1OT1. The subgroups were 0–50 vs. 50–100 [9]. The prognosis type is OS. The data were miRNAseq data from the level 3 BCGSC miRNA profiling in the TCGA LUAD project and the RNAseq data in the level 3 HTSeq-FPKM format from the TCGA LUAD project [14,25]. The supplementary data were the prognostic data from the references [29]. We used the Draw Kaplan–Meier (KM) plotter to plot the KM curve [30].

### 2.12. QRT-PCR

Our laboratory preserved the LUAD cell lines, the A549 and PC9 cells, as well as the human normal lung epithelial cell line BEAS-2B, for use in this study. To identify the DMRT3 levels in the A549, PC9, and BEAS-2B cell lines, a qRT-PCR was performed, according to [9,27]. The primer sequences used are as follows. GAPDH, Forward: CGACAGTCAGCCGCATCTTC, Reverse: CGTTCTCAGCCTTGACGGTG; DMRT3, Forward: 5′-GGTTCTCCTCGGCCCAGA-3′, Reverse: 5′-GCCGATCGGACGGGCGT-3′.

### 2.13. Statistical Analysis

An unpaired t-test was used to compare the differences between the two groups, and the data were expressed as mean ± standard deviation. A Spearman rank correlation coefficient was used to assess the correlation between the two groups. The Kaplan–Meier method was used to assess the relationship between patient prognosis and the DMRT3 expression levels, and *p* < 0.05 was considered a statistically significant difference.

## 3. Results

### 3.1. Gene Expression Analysis of DMRT3

**Figure 1:**
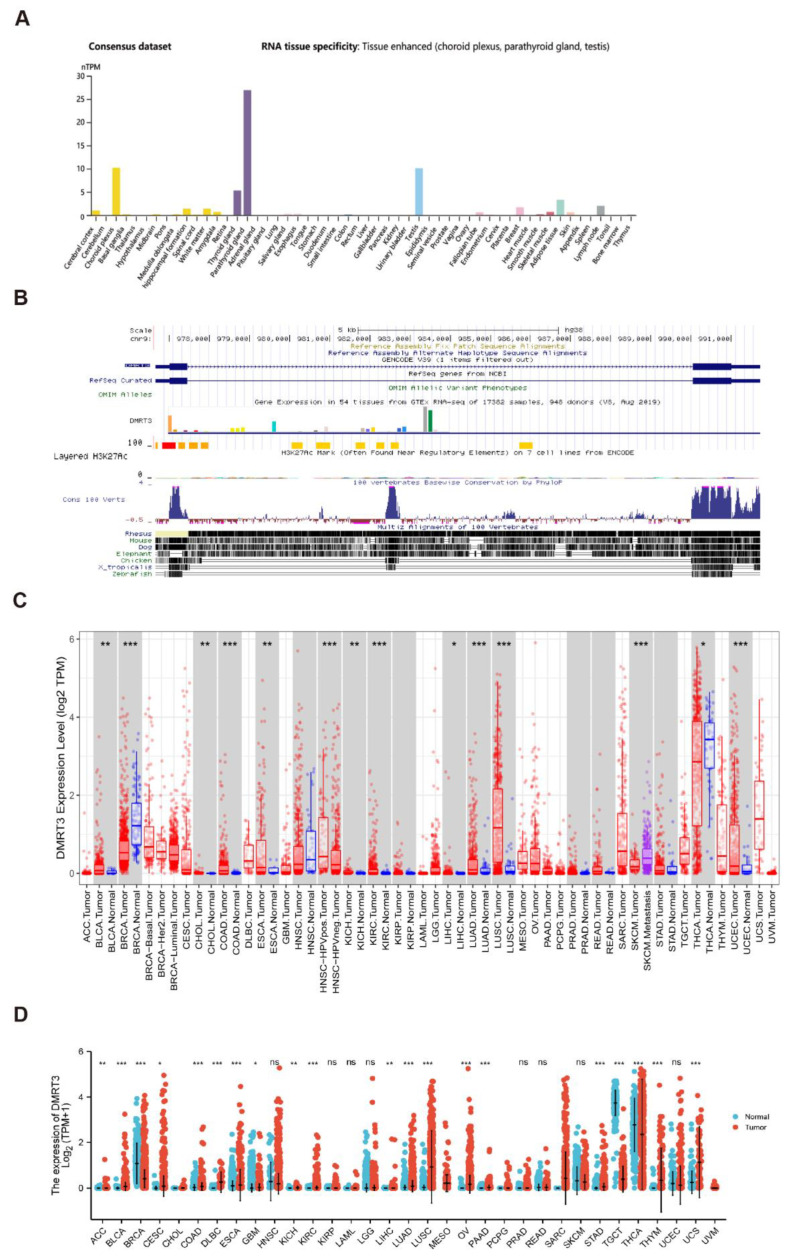
An analysis of the expression status of DMRT3 in tumors and normal tissues, as well as the conservation of the DMRT3 gene in vertebrates. (**A**) DMRT3 expression in pan-cancer (HPA database). (**B**) Visualization of the DMRT3 gene conserved analysis in vertebrates (UCSC database). (**C**) TIMER2 showing the expression status of DMRT3 in pan-cancer. (**D**) DMRT3 expression in pan-cancer (TCGA and GTEX database). Ns, *p* > 0.05; * *p* < 0.05; ** *p* < 0.01; *** *p* < 0.001.

Based on the HPA dataset, we found that DMRT3 was highly expressed in tissues, including choroid plexus, parathyroid gland, and testis (Figure 1A). The DMRT3 expression has a low tissue specificity. DMRT3 is relatively conserved in vertebrates (Figure 1B). A low tumor specificity was also observed for the distribution of DMRT3 mRNA in normal tissues. We searched the TIMER database for the mRNA expression of DMRT3 in human pan-carcinomas. DMRT3 was significantly more highly expressed in BLCA (bladder urothelial carcinoma), CHOL (cholangiocarcinoma), COAD (colon adenocarcinoma), ESCA (esophageal carcinoma), KICH (kidney chromophobe), KIRC (kidney renal clear cell carcinoma), LIHC (liver hepatocellular carcinoma), LUAD (lung adenocarcinoma), and LUSC (lung squamous cell carcinoma), and was significantly less expressed in BRCA (breast invasive carcinoma), SKCM (skin cutaneous melanoma), THCA (thyroid carcinoma), and UCEC (uterine corpus endometrial carcinoma) (Figure 1C). Next, the RNA-seq data from the TCGA and GTEX databases were used to further analyze the expression of DMRT3 in pan-cancer. The significant differences in the DMRT3 expression were found in 33 cancers, except for cancers for which no normal tissue data were available. DMRT3 was significantly more highly expressed in ACC (adrenocortical carcinoma), BLCA, CESC (cervical squamous cell carcinoma and endocervical adenocarcinoma), COAD, DLBC (lymphoid neoplasm diffuse large B-cell lymphoma), ESCA, KICH, KIRC, LIHC, LUAD, LUSC, OV (ovarian serous cystadenocarcinoma), PAAD (pancreatic adenocarcinoma), STAD (stomach adenocarcinoma), THYM (thymoma), and UCS (uterine carcinosarcoma), and was significantly less expressed in BRCA, GBM (glioblastoma multiforme), TGCT (testicular germ cell tumors), and THCA (thyroid carcinoma), compared to the corresponding normal tissues (Figure 1D). Taken together, DMRT3 was aberrantly expressed in some tumors, compared with adjacent normal tissues, and DMRT3 may be associated with the development of these tumors.

### 3.2. Relationship between the DMRT3 Expression and the Prognosis of Cancer Patients

**Figure 2:**
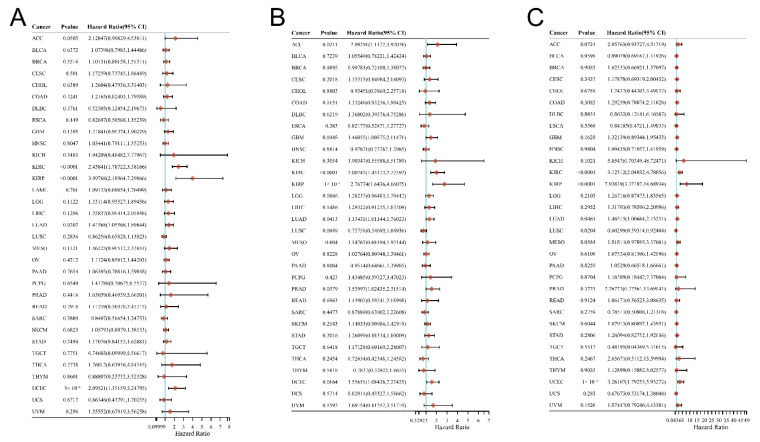
Correlation of the DMRT3 expression in pan-cancerous tumors with prognosis (forest plot). (**A**) OS, (**B**) PFS, and (**C**) DSS. Results of a single-factor Cox analysis of the individual genes in multiple tumors, including the *p* value, risk factor, HR, and confidence interval.

The relationship between the DMRT3 expression and prognosis in pan-cancer was analyzed using the univariate Cox regression analysis. As shown in Figure 2A, a high expression of DMRT3 was significantly associated with a poor OS in KIRC (HR: 2.46; 95% CI: 1.79–3.38; *p* < 0.0001), KIRP (HR: 4.00; 95% CI: 2.19–7.30; *p* < 0.0001), LUAD (HR: 1.42; 95% CI: 1.06–1.90; *p* = 0.0202), and UCEC (HR: 2.10; 95% CI: 1.35–3.25; *p* < 0.0001). As shown in Figure 2B, a high expression of DMRT3 was significantly associated with a poor PFS in ACC (HR:2.09; 95% CI: 1.12–3.92; *p* = 0.021), GBM (HR: 1.46; 95% CI: 1.01–2.11; *p* = 0.045), KIRC (HR: 2.01; 95% CI: 1.45–2.78; *p* < 0.0001), KIRP (HR: 2.77; 95% CI: 1.64–4.66; *p* < 0.0001), LUAD (HR: 1.33; 95% CI: 1.01–1.76; *p* < 0.0001), PRAD (HR: 1.54; 95% CI: 1.02–2.32; *p* = 0.038), and UCEC (HR: 1.56; 95% CI: 1.08–2.23; *p* = 0.016). As shown in Figure 2C, a high expression of DMRT3 was significantly associated with a poor DSS in KIRC (HR: 3.12; 95% CI: 2.04–4.78; *p* < 0.0001), KIRP (HR: 7.94; 95% CI: 3.37–18.69; *p* < 0.0001), LUAD (HR: 1.47; 95% CI: 1.01–2.13; *p* = 0.047), and UCEC (HR: 3.26; 95% CI: 1.79–5.93; *p* < 0.0001).

### 3.3. DMRT3 Expression between the Different Clinical Characteristics

**Figure 3:**
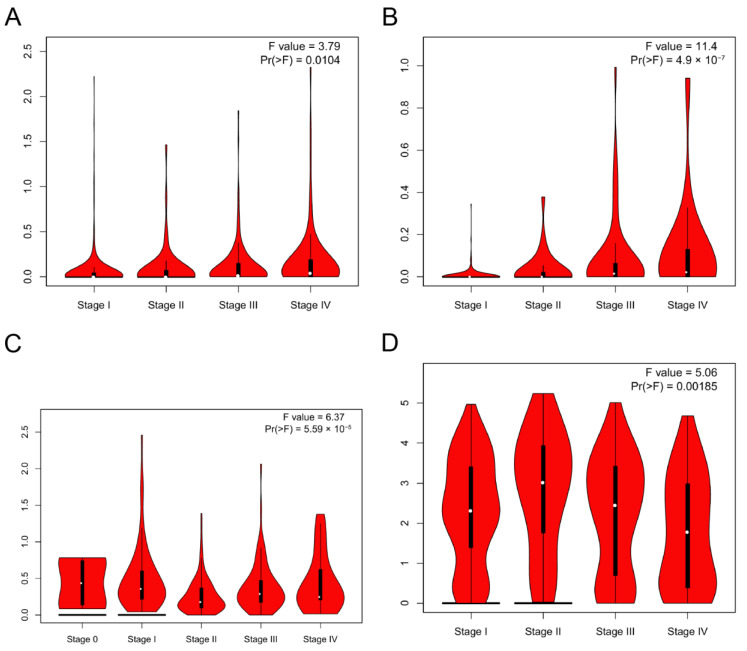
Correlation between the expression of DMRT3 in pan-cancer tumors and the pathological stage. (**A**) KIRC, (**B**) KIRP, (**C**) SKCM, and (**D**) THCA.

**Figure 4:**
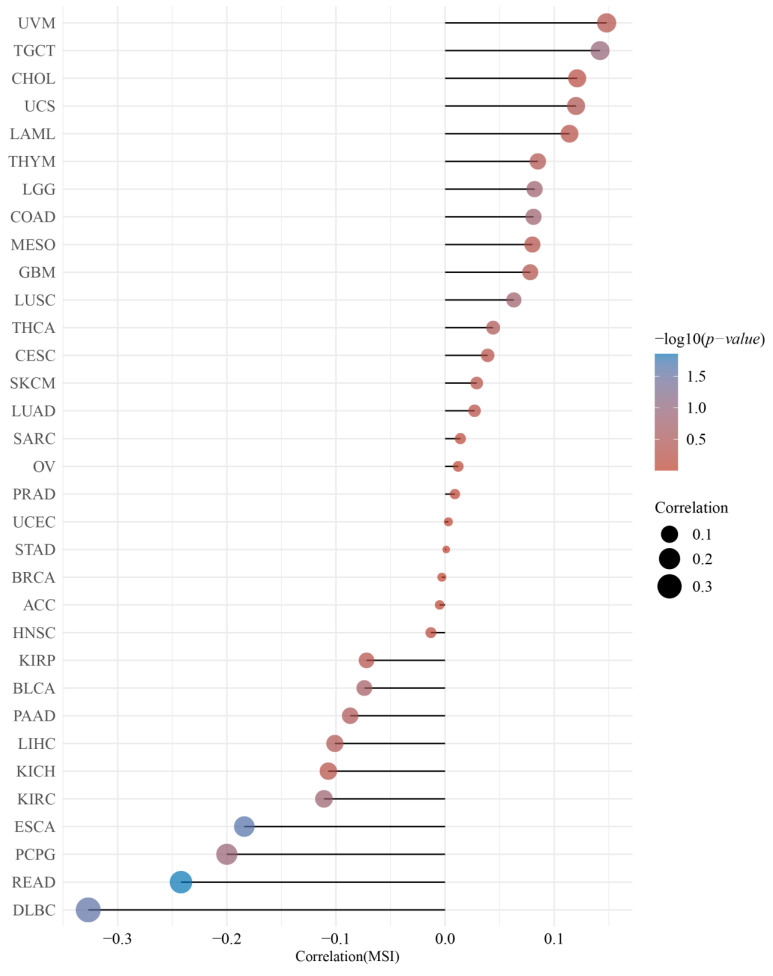
Correlation between the DMRT3 expression and microsatellite instability (MSI) in pan-cancer tumors (Spearman analysis). The horizontal coordinates represent the correlation coefficients between the DMRT3 gene and the MSI, the vertical coordinates are different tumors, the size of the dots in the figure represents the correlation coefficients, and the color of the dots represents the significance of the *p*-value, with blue representing a smaller *p*-value.

**Figure 5:**
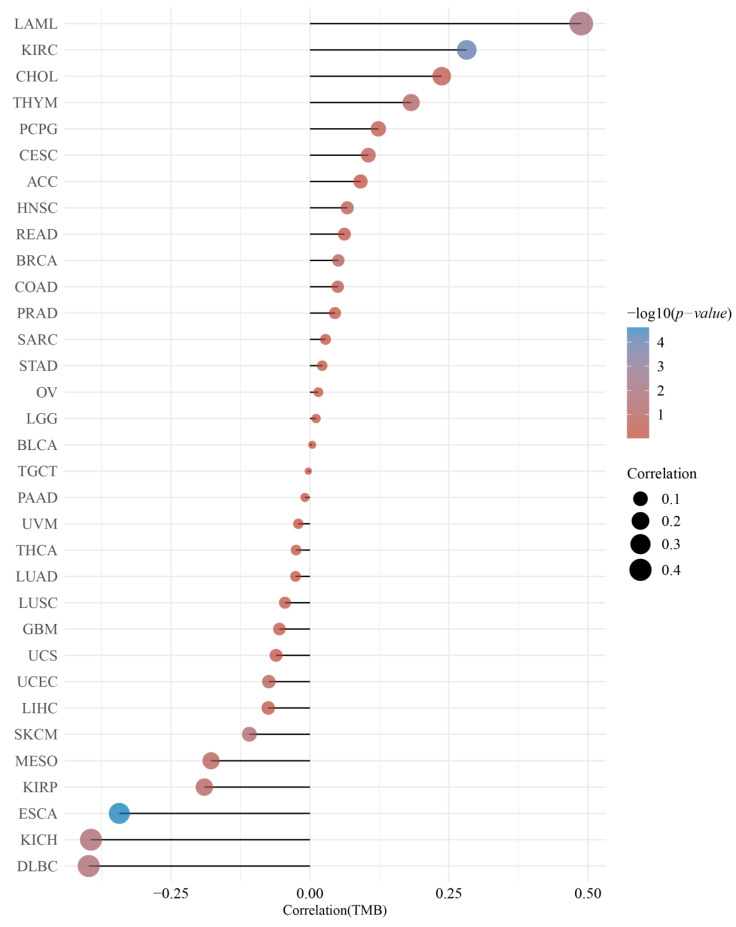
Correlation between the DMRT3 expression and the tumor mutational load (TMB) in pan-cancer tumors (Spearman analysis). The horizontal coordinates represent the correlation coefficients between DMRT3 and TMB, the vertical coordinates are for different tumors, the dot sizes represent the correlation coefficients, and the colors represent the significance of the *p*-values, with blue representing a smaller *p*-value.

GEPIA2 was used to investigate the relationship between the DMRT3 expression and tumor pathological stage. A high expression of DMRT3 was significantly associated with the advanced stages of KIRC, KIRP, SKCM, and THCA (Figure 3). The MSI occurs when some cells perform one or two alleles and a varying number of repeats and has been identified to correlate with clinicopathological features of cancer patients [31]. The TMB is the number of non-synonymous mutations per coding region of the tumor genome, including the somatic variants per megabase (MB) of the genome, identified by the reported algorithm for the whole exome sequencing [32]. As shown in Figure 4, the DMRT3 expression was negatively correlated with the MSI status of DLBC (*p* = 0.026), ESCA (*p* = 0.023), and READ (*p* = 0.013). As shown in Figure 5, the DMRT3 expression was positively correlated with the TMB status of KIRC (*p* < 0.001) and LAML (*p* = 0.009), and negatively correlated with the TMB status of DLBC (*p* = 0.017), ESCA (*p* < 0.001), KICH (*p* = 0.016), and SKCM (*p* = 0.020). Overall, these results identify DMRT3 as a potential prognostic biomarker for various cancer types.

### 3.4. The Landscape of the Genetic Alterations in DMRT3 in Different Tumors

**Figure 6:**
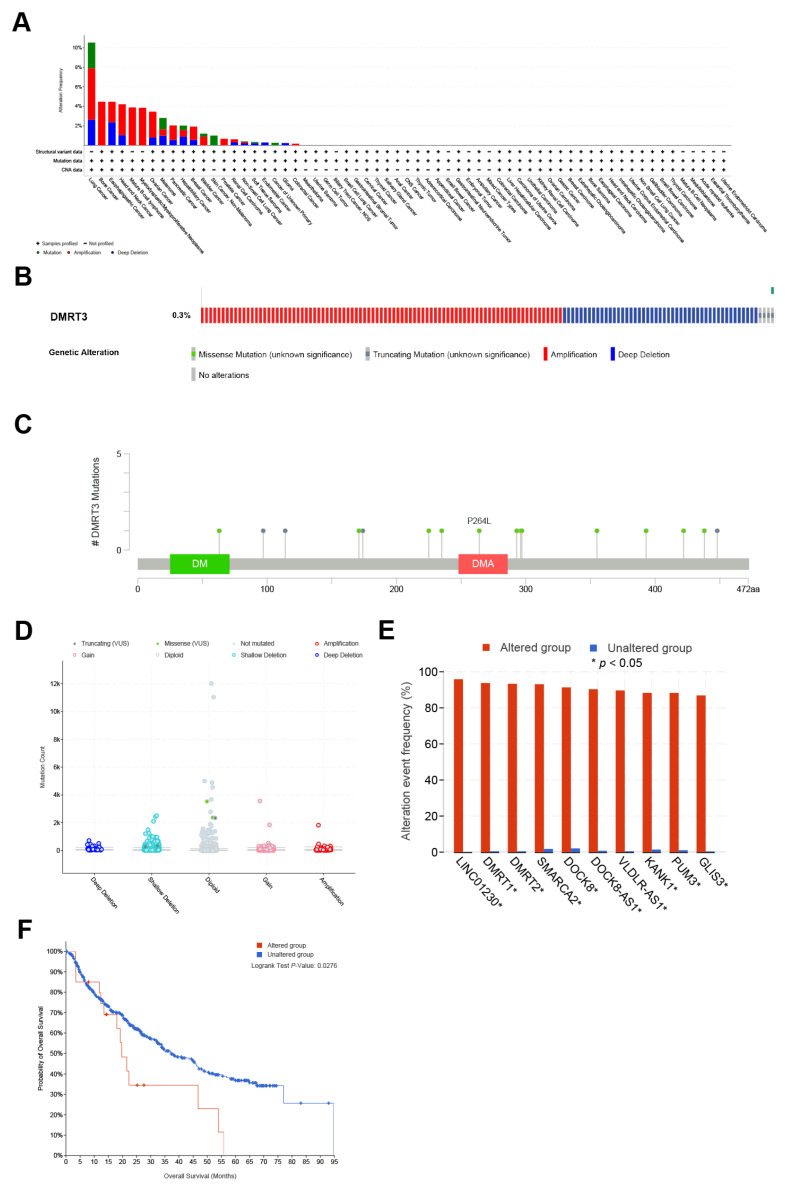
Genetic alterations in DMRT3. (**A**) Summary of the DMRT3 changes in the TCGA pan-cancer dataset. (**B**) Structure variants, mutations, and copy number alterations in DMRT3. (**C**) A description of the types, number, and location of the mutations in the DMRT3 gene. (**D**) DMRT3 alterations in pan-cancer. (**E**) Frequency of the gene alterations associated with the DMRT3 altered and unaltered groups. (**F**) Overall survival between DMRT3 altered group and DMRT3 unaltered group in pan cancer.

Using the cBioPortal database, the highest rates of genetic alterations in DMRT3 were found in patients with pan-cancer, including lung cancer (mutation 2.63%; amplification 5.26%; deep deletion 2.63%), bone cancer (amplification 4.46%), esophagogastric cancer (amplification 2.1%; deep deletion 2.36%), head and neck cancer (amplification 3.16%; deep deletion 1.05%), mature b-cell lymphoma (amplification 3.88%), myelodysplastic/myeloproliferative neoplasms (amplification 3.85%), ovarian cancer (amplification 2.65%; deep deletion 0.8%), melanoma (mutation 1.16%; amplification 0.66%; deep deletion 0.99%), pancreatic cancer (amplification 1.49%; deep deletion 0.55%), hepatobiliary cancer (mutation 0.45%; amplification 0.67%; deep deletion 0.9%), breast cancer (amplification 1.36%; deep deletion 0.56%), bladder cancer (mutation 0.3%; amplification 0.9%), skin cancer, non-melanoma (mutation 1%), prostate cancer (amplification 0.67%), renal cell carcinoma (amplification 0.31%; deep deletion 0.31%), non-small cell lung cancer (mutation 0.07%; amplification 0.14%; deep deletion 0.21%), soft tissue sarcoma (mutation 0.11%; deep deletion 0.23%), endometrial cancer (amplification 0.29%), cancer of an unknown primary (deep deletion 0.26%), glioma (deep deletion (0.24%), and colorectal cancer (amplification 0.17%) (Figure 6A). The amplification, deep deletion, missense mutations, and truncating mutations, were the main types of frequent genetic alterations in DMRT3 (Figure 6B). The types, loci, and number of cases of the DMRT3 gene modifications were further shown (Figure 6C), including 12 missense mutations (P235S, P438S, S171I, T293M, R297Q, E63K, S422L, E296K, A225S, N393K, A355V, and P264L) and four truncating mutations (D174Lfs*2, Q448*, P98Rfs*56, and Q114*). The most common frequent putative copy number alterations for DMRT3 were amplification, gain, diploid, and shallow deletion (Figure 6D). As a result of the gene alterations in LINC01230, DMRT1, DMRT2, SMARCA2, DOCK8, DOCK8-AS1, VLDLR-AS1, KANK1, PUM3, and GLIS3, the altered group was more likely to be affected than the unaltered group (Figure 6E). As shown in Figure 6F, the OS was significantly lower in the DMRT3 altered group (n = 23) than in the DMRT3 unaltered group (*n* = 508) (*p* = 0.0276).

### 3.5. DMRT3 Gene Expression in the Pan-Cancer Tumors Correlates with the Immune Infiltration

**Figure 7:**
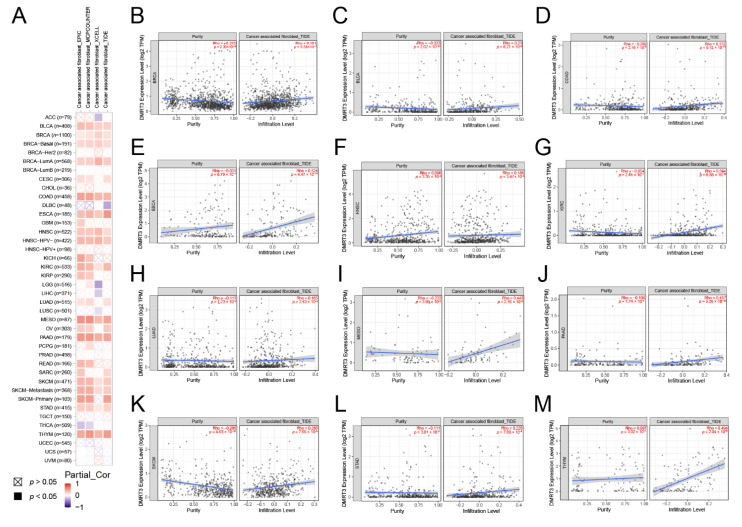
Correlation of the DMRT3 expression with cancer-associated fibroblasts (CAF) in pan-cancer tumors. (**A**) Correlation analysis between the DMRT3 expression and CAF was performed using TIMER2.0. (**B**) BLCA. (**C**) BRCA. (**D**) COAD. (**E**) ESCA. (**F**) HNSC. (**G**) KIRC. (**H**) LUAD. (**I**) MESO. (**J**) PAAD. (**K**) SKCM. (**L**) STAD. (**M**) THYM.

The tumor microenvironment (TME) is made up of four main components: the immune component, the vascular component, the extracellular matrix, and the stromal component, providing the crosstalk between the tumor cells and the microenvironment. There are many immune cells in the immune component, including T cells and B cells, while the stromal component consists of cancer-associated fibroblasts and mesenchymal stem cells [33]. The TIMER 2.0 website was used to analyze the correlation between the DMRT3 expression and TME components in pan-cancer. As shown in Figure 7A, we obtained some results by the EPIC, MCPCOUNTER, XCELL, and TIDE algorithms, and the DMRT3 expression in some tumors was positively correlated with CAF, and the DMRT3 expression in some tumors was positively correlated with CAF. As shown in Figure 7B–M, the results of the TIDE algorithm showed that the expression of DMRT3 in BLCA, BRCA, COAD, ESCA, HNSC, KIRC, LUAD, MESO, PAAD, SKCM, STAD, and THYM was significantly and positively correlated with CAF.

### 3.6. DMRT3 Gene Expression in Pan-Cancer Tumors Correlates with the Immune Checkpoint Genes

**Figure 8:**
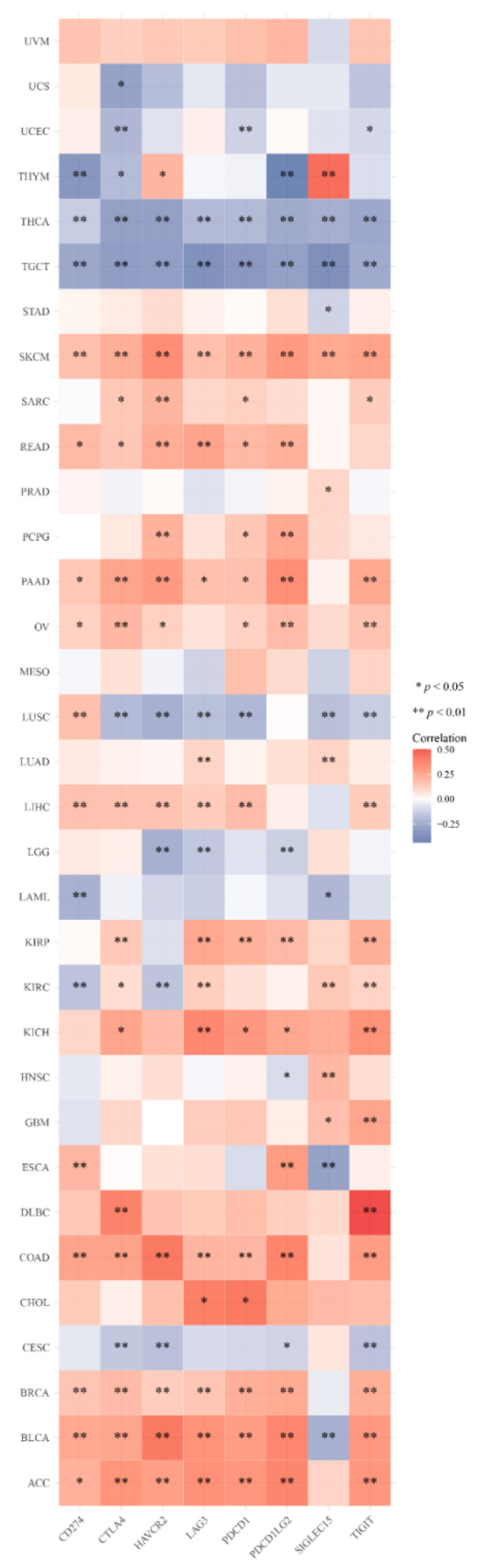
Expression of DMRT3 in the pan-cancer species was associated with the immune checkpoint genes. Heat map of the expression of the immune checkpoint-related genes in different tumor tissues, where the horizontal coordinates represent different immune checkpoint genes, the vertical coordinates represent different tumor tissues, and each box in the figure represents the correlation analysis between the expression of the DMRT3 gene and the expression of the immune checkpoint-related genes in the corresponding tumor, the different colors represent the changes in the correlation coefficients.

In recent years, tumor immunotherapy has attracted a great deal of attention. We evaluated the correlation between the DMRT3 expression and eight immune checkpoint genes. The results show that the DMRT3 expression levels correlate significantly with the expression of the immune checkpoint genes in 31 tumors. DMRT3 was significantly correlated, positively or negatively, with the expression of the immune checkpoint genes in five tumor types, including BLCA, ESCA, KIRC, LUSC, and THYM. The results showed that the expression of DMRT3 correlated negatively with the expression of the immune checkpoint genes in 18 tumor types, including ACC, BRCA, CHOL, COAD, DLBC, GBM, HNSC, KICH, KIRP, LIHC, LUAD, OV, PAAD, PCPG, PRAD, READ, SARC, and SKCM (Figure 8). The results showed that the expression of DMRT3 correlated positively with the expression of the immune checkpoint genes in eight tumor types, including CESC, LAML, LGG, STAD, TGCT, THCA, UCEC, and UCS (Figure 8). These results suggest a potential role for DMRT3 in influencing the tumor immunity by regulating these immune checkpoint genes. 

### 3.7. Biological Function of DMRT3 in Cancer

**Figure 9:**
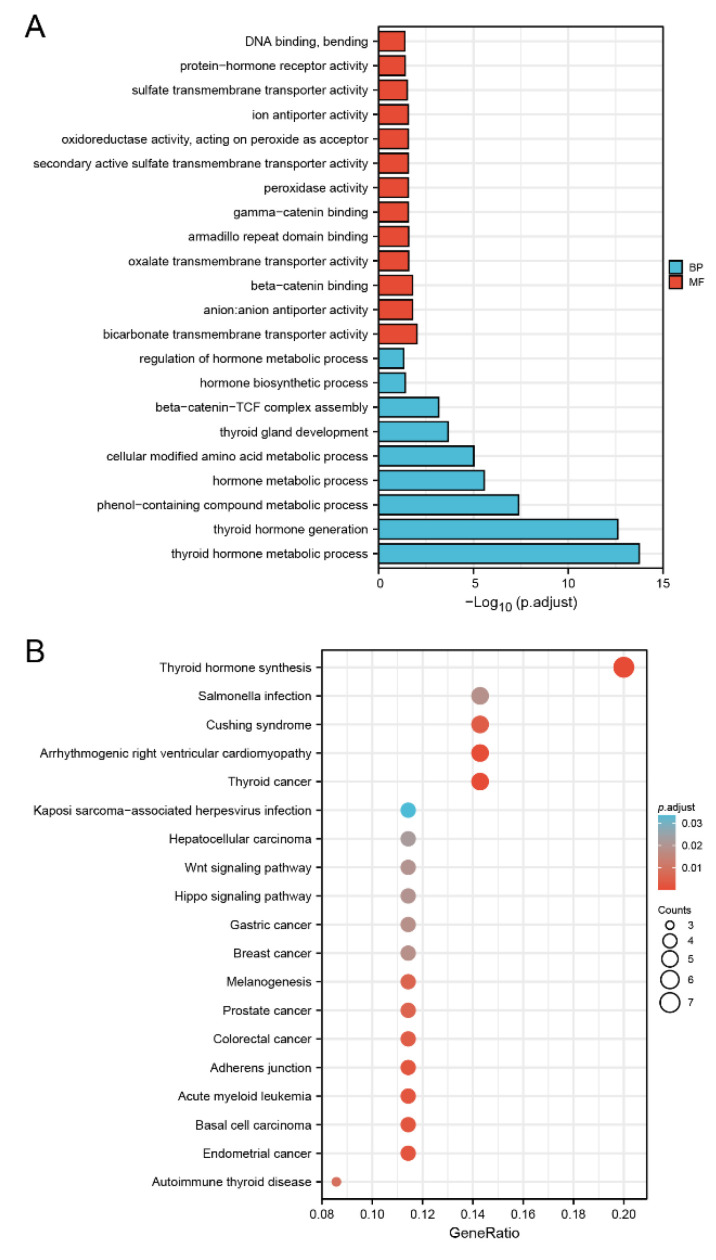
Gene ontology (GO) and Kyoto Encyclopedia of Genes and Genomes (KEGG) analysis of the top 100 genes co-expressed with DMRT3, obtained by GEPIA2. (**A**) GO analysis of the DMRT3 co-expressed genes. (**B**) KEGG analysis of the DMRT3 co-expressed genes.

To investigate the possible mechanisms of the DMRT3 action in pan-cancer, we extracted the top 100 genes with expression patterns similar to DMRT3, using GEPIA2 from the TCGA dataset, and obtained 16 genes co-expressed with DMRT3 using the STRING tool. We performed GO and KEGG analyses on these 116 genes. As shown in Figure 9A, the results showed five significantly related biology processes, including the thyroid hormone metabolic process, thyroid hormone generation, phenol-containing compound metabolic process, hormone metabolic process, and cellular modified amino acid metabolic process, as well as five significantly related molecular functions, including bicarbonate transmembrane transporter activity, anion: anion antiporter activity, beta-catenin binding, oxalate transmembrane transporter activity, and armadillo repeat domain binding. As shown in Figure 9B, five pathways including the thyroid hormone synthesis, thyroid cancer, arrhythmogenic right ventricular cardiomyopathy, endometrial cancer, and basal cell carcinoma, may be the main pathways involved in the development of pan-cancer by DMRT3.

### 3.8. Drug Sensitivity Analysis of DMRT3

We further investigated the potential correlation between drug sensitivity and the DMRT3 expression using the RNAactDrug database. The DMRT3 expression was positively correlated with the drug sensitivity of tanespimycin, PD-0325901, PLX4720, trametinib, and refametinib (Table 1). The DMRT3 expression was negatively correlated with the drug sensitivity of GSK690693, PHA-793887, NPK76-II-72-1, TAK-715, and PI-103 (Table 1). These data suggest that DMRT3 may be associated with a chemoresistance to certain chemotherapeutic agents. 

### 3.9. Cancer-Related Expression Pattern of DMRT3 in Single Cells

**Figure 10:**
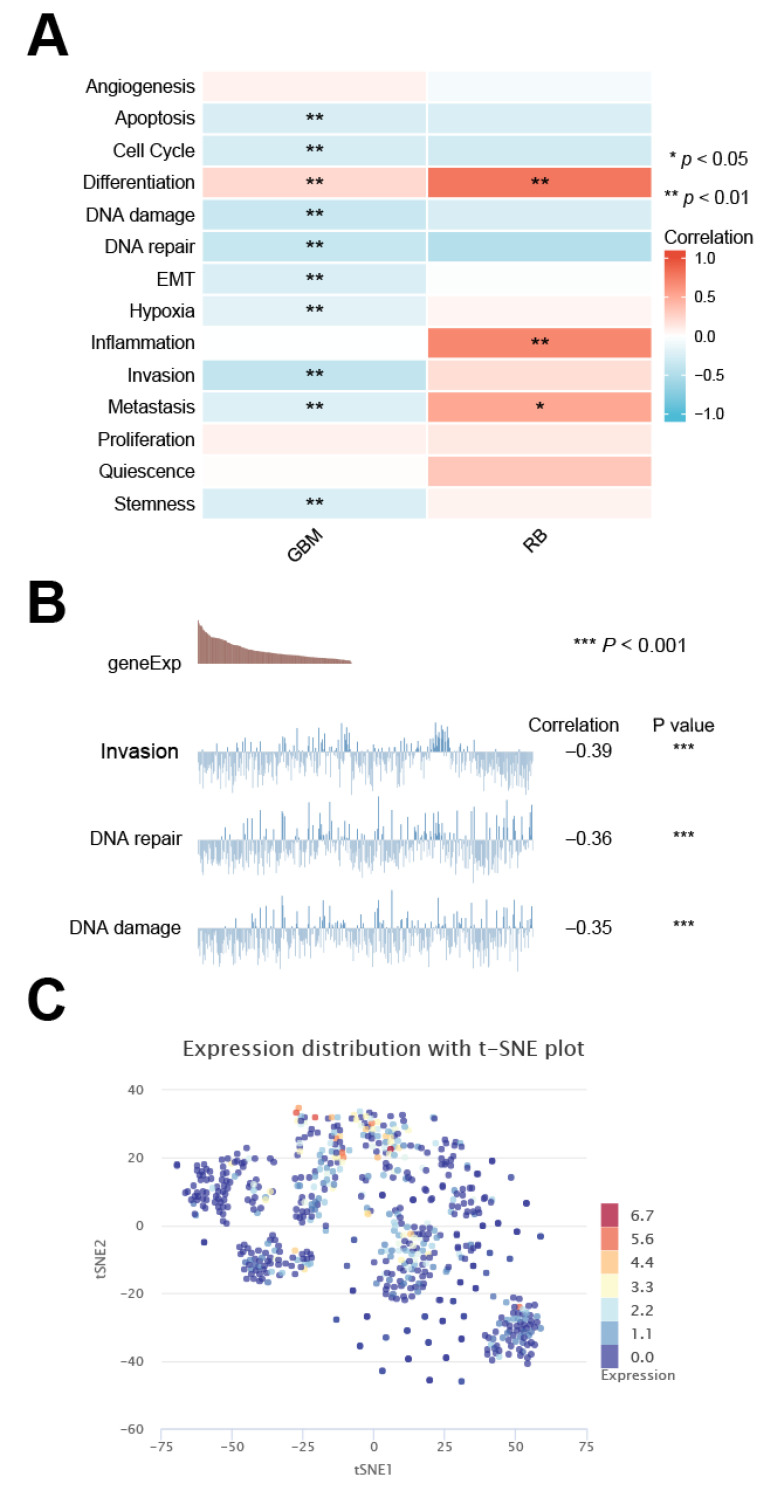
Single cell sequencing of DMRT3 and its relationship to the tumor functional status. (**A**) DMRT3 expression, compared with different tumor functional status, based on the CancerSEA database (**B**) Correlation between the DMRT3 expression, based on the CancerSEA database and three significantly different functional statuses. (**C**) T-SNE plots showing the DMRT3 expression profiles in the single cells of the GBM samples.

The DMRT3 expression in GBM was significantly positively correlated with differentiation, and significantly correlated with the apoptosis, cell cycle, DNA damage, DNA repair, EMT, hypoxia, invasion, metastasis, and stemness; and the DMRT3 expression in RB was significantly positively correlated with the differentiation, inflammation, and metastasis (Figure 10A). As shown in Figure 10B, the results showed the relationship between the DMRT3 expression and DNA repair, the DMRT3 expression and DNA damage, and the DMRT3 expression and invasion in GBM. The T-SNE plots showed the expression profile of DMRT3 in the GBM single cells (Figure 10C). According to these results, DMRT3 may be involved in the progression of cancer.

### 3.10. CeRNA Network of DMRT3

**Figure 11:**
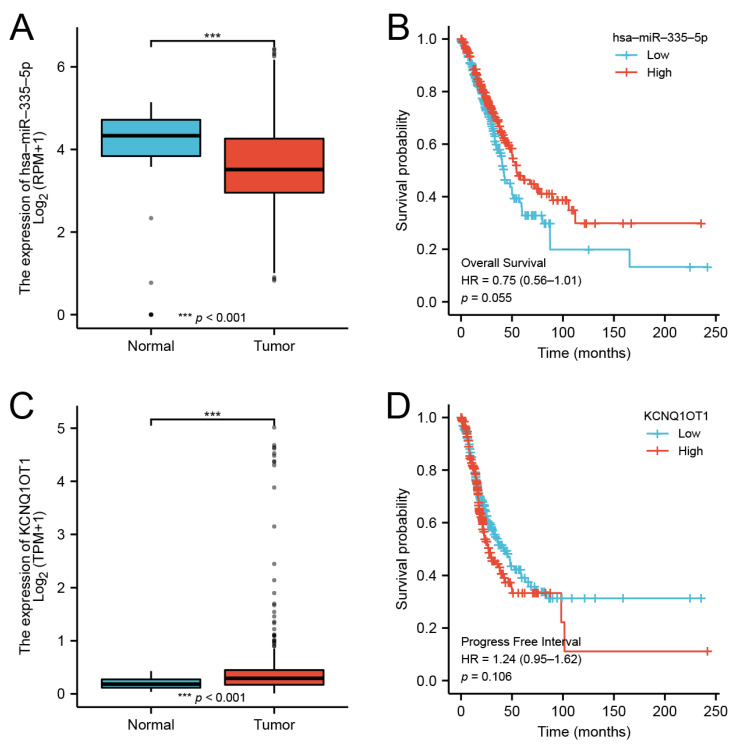
Expression and prognosis of the DMRT3 ceRNA network in LUAD. (**A**) Expression of the miR-335-5p in LUAD and unpaired normal tissues. (**B**) Prognosis of the miR-335-5p in LUAD. (**C**) Expression of the KCNQ1OT1 in LUAD and unpaired normal tissues. (**D**) Prognosis of the KCNQ1OT1 in LUAD.

**Figure 12:**
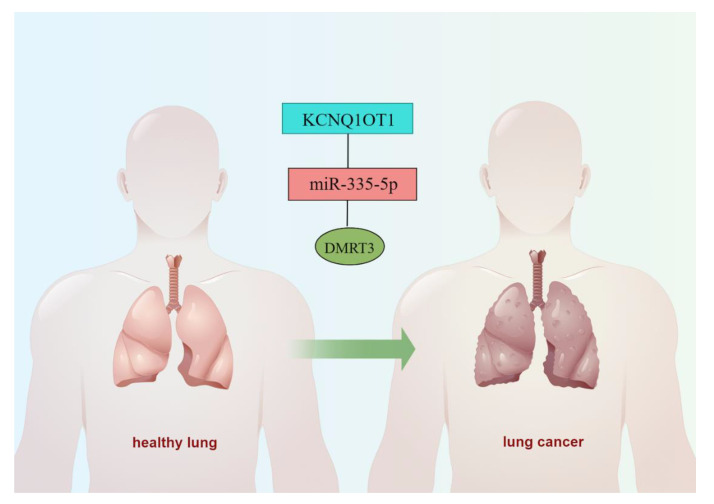
A possible ceRNA network for the DMRT3-mediated development of LUAD.

Our findings suggested that DMRT3 was up-regulated in most tumor types. However, there are no studies investigating the upstream regulatory mechanisms of DMRT3. Through a database analysis, we found that DMRT3 may be a target of miR-335-5p, while miR-335-5p may be a target of KCNQ1OT1. In LUAD, miR-335-5p was lowly expressed in LUAD (Figure 11A) and the low expression of miR-335-5p suggested a poor prognosis (Figure 11B). KCNQ1OT1 was highly expressed in LUAD (Figure 11C) and the expression of KCNQ1OT1 suggested a poor prognosis (Figure 11D). Therefore, we constructed a ceRNA network of KCNQ1OT1/miR-335-5p/DMRT3 in LUAD (Figure 12).

### 3.11. Validation of the DMRT3 Expression in the Cell Lines

**Figure 13:**
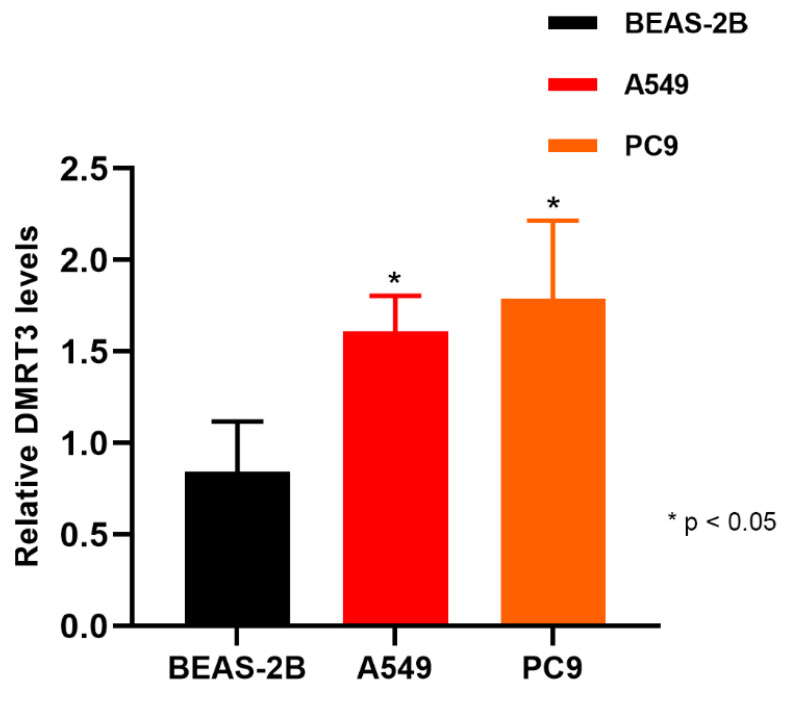
Expression of DMRT3 in A549, PC9, and Beas-2B.

The expression of DMRT3 in A549 was significantly higher than that in BEAS-2B (1.610 ± 0.191 vs. 0.842 ± 0.275, *p* = 0.0311) (Figure 11). The expression of DMRT3 in PC9 was significantly higher than that in IOSE29 (1.789 ± 0.427 vs. 0.842 ± 0.275, *p* = 0.0268) (Figure 13). These results suggested that DMRT3 was significantly upregulated in the LUAD cell lines, compared with the lung epithelial cells.

## 4. Discussion

Using pan-cancer analyses, we are able to better understand the underlying molecular abnormalities of various cancers and to find biomarkers for early detection and the targeted treatment of cancer. TCGA analyzes 33 common tumor types using multi-omics data, offering the opportunity to uncover molecular aberrations across cancer types [34,35]. 

DMRT3 is aberrantly expressed in pancreatic ductal carcinoma, CRC, and LUAD [36,37,38]. However, there is a paucity of research on DMRT3 in other cancer types. Therefore, in this study, we have focused our research on the role of DMRT3 in pan-cancer. In the present study, DMRT3 was found to be aberrantly expressed in most tumors, compared with the adjacent normal tissues. DMRT3 was significantly upregulated in the LUAD cell lines.

In addition, we attempted to analyze the correlation between the DMRT3 mRNA expression levels and prognosis in pan-cancer. A high expression of DMRT3 is significantly associated with a poor OS in KIRC, KIRP, LUAD, and UCEC. A high expression of DMRT3 is significantly associated with a poor PFS in ACC, GBM, KIRC, KIRP, LUAD, and UCEC. A high expression of DMRT3 is significantly associated with a poor DSS in KIRC, KIRP, LUAD, LUSC, and UCEC. In summary, these findings suggest that DMRT3 may be a useful predictor of cancer prognosis for practical applications.

However, there are no relevant studies on the DMRT3 gene alterations in human cancers. Using the cBioPortal database, we discovered that amplification is the most common DMRT3 alteration in pan-cancer. The co-occurrence of LINC01230, DMRT1, DMRT2, SMARCA2, DOCK8, DOCK8-AS1, VLDLR-AS1, KANK1, PUM3, and GLIS3 alterations was found to be observed in the DMRT3 alteration group.

In this study, we first showed evidence of a potential association between the DMRT3 expression and staging, the MSI or TMB. The results showed that a high expression of DMRT3 in four tumors was significantly associated with the advanced tumor stage. Further analysis showed that the DMRT3 expression was significantly associated with the MSI in three cancer types and with the TMB in six cancer types. According to these results, the DMRT3 expression may affect cancer patients’ responses to immunocheckpoint therapy, which will contribute to a better understanding of immunotherapy mechanisms for cancer treatment.

Researchers are currently focusing on the tumor microenvironment (TME). CAF plays various pro-tumorigenic functions during tumorigenesis, as a major component of the TME [33]. Therefore, we suspect that DMRT3 may also be involved in the immune regulation of human cancers. According to the results, DMRT3 may be involved in the immune escape in human cancer immunotherapy, and the TIMER database mining further revealed that a correlation between the DMRT3 expression and the level of infiltration of various immune-related cells. In this study, our correlation analysis showed that the DMRT3 expression in many tumor types was significantly correlated with the expression of eight immune checkpoint genes. To date, little is known about the role of DMRT3 in the human immune system, and the role of DMRT3 in the tumor immune microenvironment needs to be further investigated. As a result of our research, we have elucidated the potential role of DMRT3 in the tumor immunity and its prognostic value.

DMRT3 and OAS3 are involved in human disorders of sex development (DSD) through the control of the ESR1 expression [39]. DMRT3/5, together with EMX2, localizes the pallidophore-hypopallidophore boundary by antagonizing the ventral homologous frame transcription factor GSX2 [40]. DMRT3 is also a transcription factor associated with lung squamous carcinoma heterogeneity and, together with TP63/SOX2, regulates the genes involved in epithelial cell differentiation and is known as an oncogenic transcription factor [38]. Several genes co-expressed with DMRT3, in tumors and other tissues, were identified using STRING and GEPIA2. By a KEGG analysis, we found that DMRT3 was involved in the most common signaling pathways in pan-cancer, including thyroid hormone synthesis, thyroid cancer, arrhythmogenic right ventricular cardiomyopathy, endometrial cancer, and basal cell carcinoma. These findings set the stage for further exploration of the molecular function of DMRT3. 

It is unknown whether the DMRT3 expression is associated with a drug sensitivity or resistance. However, we found that it was associated with several sensitivities using the RNAactDrug database, including tanespimycin, PD-0325901, PLX4720, GSK690693, PHA-793887, and NPK76-II-72-1. Therefore, we hypothesize that DMRT3 may play a role in chemotherapy and may be associated with chemoresistance.

The DMRT3 expression in GBM was significantly positively correlated with the differentiation and negatively correlated with the differentiation, inflammation, and metastasis. The specific regulatory mechanisms of DMRT3 in GBM require further study. Our study has some limitations. Firstly, the relatively small sample sizes of some of the less common tumor types may lead to batch effects or inaccurate results. Secondly, the study demonstrates only the preliminary findings linking DMRT3 to cancer progression in various types of tumors, and more experiments are required to determine its precise molecular function in tumorigenesis. We constructed a ceRNA network of KCNQ1OT1/miR-335-5p/DMRT3 in LUAD. The findings in this paper are based on the bioinformatics analysis without experimental data. Further research is needed to verify our findings. The role of DMRT3 in cancer could be better understood with a large sample size and comprehensive analysis of DMRT3.

## 5. Conclusions

There are a number of cancers where DMRT3 can be a potential prognostic factor. A number of pathways are modulated by DMRT3, including the MSI, TMB, cancer-associated fibroblast infiltration, immune checkpoint inhibitors, and drug sensitivity. A multifaceted role of DMRT3 in pan-cancer is highlighted in this study, providing a rationale for the use of DMRT3 as a new therapeutic approach.

## Figures and Tables

**Table 1:** Drug sensitivity analysis of DRMT3.

Compound	RNA Molecule	Source	Spearman.Stat	Spearman.Fdr	*p* Value
Tanespimycin	DMRT3	GDSC	0.194987316	8.98 × 10^−9^	1.33 × 10^−9^
PD-0325901	DMRT3	CCLE	0.164608073	0.044844396	0.00526032
PLX4720	DMRT3	CCLE	0.16154357	0.043031472	0.006181571
Trametinib	DMRT3	GDSC	0.160737175	2.58 × 10^−6^	6.26794 × 10^−7^
Refametinib	DMRT3	GDSC	0.154929636	6.85 × 10^−6^	1.58274 × 10^−6^
GSK690693	DMRT3	GDSC	−0.15192674	1.09 × 10^−5^	2.52199 × 10^−6^
PHA-793887	DMRT3	GDSC	−0.158247668	3.48 × 10^−6^	9.36157 × 10^−7^
NPK76-II-72-1	DMRT3	GDSC	−0.159673169	2.33 × 10^−6^	7.44586 × 10^−7^
TAK-715	DMRT3	GDSC	−0.159971009	2.25 × 10^−6^	7.09624 × 10^−7^
PI-103	DMRT3	GDSC	−0.174534416	4.26 × 10^−7^	6.06437 × 10^−8^

## Data Availability

All data generated or analyzed during this study are included in this article.
